# Trend in distribution of primary health care professionals in Jiangsu province of eastern China

**DOI:** 10.1186/s12939-014-0117-z

**Published:** 2014-11-28

**Authors:** Kang Xu, Kaijin Zhang, Dan Wang, Ling Zhou

**Affiliations:** Department of Medical Administration, Huai’an First People’s Hospital, Nanjing Medical University, 6 Beijing West Road, Huai’an, Jiangsu 223300 China; School of Public Health, Southeast University, 87 Dingjia Bridge, Nanjing, Jiangsu 210009 China; Department of Epidemiology and Biostatistics, School of Public Health, Nanjing Medical University, 818 Tianyuan East Road, Nanjing, Jiangsu 211166 China

**Keywords:** Primary health care, Health workforce, Inequality, People’s Republic of China

## Abstract

**Introduction:**

Since the late 1990s, the Chinese government has carried out several reforms on the primary health care, which is greatly improved but still left much to be desired, especially for the health workforces. The aim of this study was to analyze the number of health workforces and the trends in distribution of health workforces in Jiangsu province of eastern China from 2008 to 2012.

**Methods:**

The time trends in number and distribution of health professionals were compared in study period. Lorenz curves were plotted and Gini coefficient, Atkinson index and Theil index were calculated for inequalities in the distribution of health workforces to population and area.

**Results:**

The number of health workforces increased every year and the inequality in the distribution of health workforces showed a decline trend from 2008 to 2012. After 2009, these trends changed more rapidly. There was the disproportionality between physicians and nurses. The values of three inequality indicators based on area were larger than those based on population.

**Conclusion:**

The health reform in 2009 might play an important role in increasing the number of health workforces and improving the distribution of health workforces in primary health care facilities. The disproportionality between physicians and nurses was related to the shortage of number of nurses.

## Introduction

Primary health care (PHC) system is the key component of almost any health system in the world. Large numbers of researches [1,2] have shown that a good PHC system not only improves population health, but also bridges the gaps in health caused by the disparities of socio-economic states. However, as a result of low health human resource capacity and underinvestment, primary health care in many low and middle-income countries has been neglected [3]. PHC has its own merits that include greater focus on preventative, improved access to basic care measures to maintain wellbeing and manage health problems before they become severe and/or life threatening [4]. For the rapid development of China, PHC is also important.

Although China has made rapid development in economy in recent years, it is still a developing country with a GDP per capita of $6076 [5] and more than 1.3 billion people [6]. China once established an enviable PHC system which was inexpensive and was a good model for other countries before market reforms in 1978. More than 90% of the population was provided with nearly universal health insurance and health care was easily accessible to patients through barefoot doctors [7]. In order to enhance the efficiency in the health system, a market-oriented reform was introduced in the health sector in 1978. Firstly, government introduced a competition among the three-tiered health service facilities to establish a competitive health service market; secondly, patients needed to pay the health service providers directly out-of-pocket. In order to get the maximum profits and survive in fierce competition market, the health facilities enlarge their scale, update their equipment, and enroll excellent physicians to attract patients. This resulted in an excessive concentration of health resources and patients at the third-tiered health institutions [8], the phenomena of a rise in medical costs, excessive use of drugs, advanced diagnostic tests and a decline in the use of PHC. Thus health disparity gap between the poor and the rich became gradually wider [9]. However, the government made a decision to establish a convenient and affordable PHC system through community health facilities in urban areas at the end of 1990s. The government hoped to take examples by the successful health care system of the countries which reduce health inequities across populations through a strong PHC system [10,11].

Even though the reforms have made PHC renewal to some extent since 1990s, the problems of ‘difficult access to medical services and expensive medical cost’ have still not been solved, and even became more serious in recent years. In addition, there have been few reports on the number of health workforces and distribution of health workforces in PHC facilities in China. Luo et al. [12] conducted a comparative study on the number of health workforces between Anhui and the nation from 2004 to 2010, but they did not evaluate the distribution of health workforce. Wang et al. [13] analyzed the number of health professionals and the distribution of health professionals in China between 2006 and 2009. They found the number of health professionals increased and the inequality in health professionals improved from 2006 to 2009. But they just evaluated parts of PHC facilities.

Jiangsu is an eastern coastal province of China, which had 79 million people in 2012. The total gross domestic product of the province was about Ұ5000 billion RMB in 2012, ranking in the second place of all provinces in China. As an eastern big province, the government of Jiangsu province has clarified its goal of establishing a strong PHC system. Human workforces in PHC system would be the key factor in determining their performance. The purpose of this study was to compare the number of health workforces and the trends in distribution of health workforces in PHC facilities in Jiangsu before and after the reform of the medical and health sectors in 2009. The results of the study would be helpful to reflect effect of the reform on the distribution of health workforces and can be references for the government to formulate health personnel development policies.

## Methods

### Setting

Jiangsu province consists of 13 cities, and each city consists of many administrative divisions such as districts, counties, towns and villages. PHC facilities spread over the administrative region at different levels. The community health service organizations (CHOs), including both health centers and health stations, mainly locate in districts and counties in urban areas, while township health centers (THCs) and village clinics distribute in towns and villages. At the same time, polyclinics and other clinics play necessary complementary role in providing PHC to students and teachers in schools, staffs in enterprise and general population. PHC facilities in China must inform the National Health and Family Planning Commission (NFHPC) every quarter about the health workforces by national health information network reporting system [14]. Both of the NFHPC and health department of every province publish data on the development of health human resources of corresponding province every year by statistical yearbook. The data about health workforces used for analysis in this study came from the compilation of health statistical data (internal materials) of Jiangsu province. Demographic and population data were from the province statistical yearbook. In the present study, health professionals consisted of practicing physicians, assistant practicing physicians, registered nurses, pharmacists, technicians and medical interns. Even though county doctors and hygienists provide PHC in rural areas, they just accepted short-term medical training, and they were not included in health professionals in yearbook. Practicing physician and assistance practicing physician were designated as physician, and registered nurse was aliased as nurse.

### Analysis

Trends in the number and the distribution of health workforces in PHC facilities were compared between 2008 and 2012, and the physicians-to-nurses ratio and the physicians-to-health professionals ratio in each year were calculated. The health workforces-to-population and health workforces-to-area ratios in each municipal administrative area of the province were analyzed.

Three indicators were calculated to evaluate inequality in the distribution of health workforces in PHC facilities, fundamentally based on the ratios of health workforces to population and area in each municipal body of the province. We plotted the Lorenz curves and calculated inequality indicators: the Gini coefficient [15], Atkinson index [16], and Theil index [17,18]. These indicators were initially used to study inequality of income or wealth, and they have been applied to analyze the distribution of health workforces such as general physician distribution [16,19]. Three different indicators would make the time trend of inequality more robust. *Distribution Analysis Stata Package (DASP)* [20] was used to calculate the inequality indices and plot Lorenz curves.

### Inequality indicators

#### Lorenz curve and gini index

The Lorenz curve is now commonly applied to evaluate the inequality in the distribution of health care resources [15,21-23]. The Lorenz curve compares the distribution of an interest variable with the uniform distribution that represents equality. This equality distribution is shown by a perfect line, and in fact it is the diagonal line. The greater the Lorenz curve lies from this line, the greater inequality the distribution of interest variable is. In this curve, the cumulative share of the population ranked by the interest variable in an increasing order is generally shown on the X axis, and the cumulative share of the interest variable on the Y axis. In our study, the X axis illustrates the cumulative percentage of population and area of each city in the province and the Y axis showed the cumulative percentage of health workforces in PHC facilities of each city.

The Gini coefficient is one of the most commonly used indicators of inequality [15,16,21], which is derived from the Lorenz curve. The Gini is equal to the ratio of the area between the Lorenz curve and the diagonal line, to the whole area below the diagonal line. The Gini coefficient ranges from zero (perfect equality) to one (maximum inequality).

#### Atkinson index

Atkinson index links a social welfare function to a concept of equality, which is one of the few inequality measures that explicitly incorporate normative judgments about social welfare [16]. The Atkinson index is derived by calculating the equity-sensitive average interest variable (*y*_*ɛ*_), which is the level of per capita health workforces that if equally distributed would give the same level of social welfare as the actual distribution of health workforces. The index is given by the following formula:

1 $$ {y}_{\varepsilon }=1-{\left\lfloor {\displaystyle \sum_i{\left({Y}_i\right)}^{1-\varepsilon }f\left({Y}_i\right)}\right\rfloor}^{1/1-\varepsilon } $$

2 $$ {A}_{\varepsilon }=1-\frac{y_{\varepsilon }}{\mu } $$

where *Y*_*i*_ is the proportion of total number of health workforces within the *i*th group, *μ* is the average of health workforce, and *ɛ* is used to assume the level of inequality aversion which reflects the strength of society’s aversion to inequality (or preference for equality). It ranges from 0 to infinity. When the parameter *ɛ* >0, it shows that there is a social aversion to inequality (or a preference for equality). As the value of *ɛ* rises, relatively more weight is attached to transfers at the lower end of the distribution, and relatively less at the upper end. The more equal the distribution of the interest variable, the closer *y*_*ɛ*_ will be to *μ* and the lower the value of the Atkinson index. The coefficient *ɛ* in present study was set at 0.5 to calculate the Atkinson indices.

#### Generalized entropy index

General entropy index involves a different conceptual basis based on the entropy concepts which is used in information theory. The index [17] is defined as

$$ GE\left(\theta \right)=\frac{1}{\theta^2-\theta}\left[\frac{1}{\mathrm{n}}{\displaystyle \sum_{i=1}^n{\left(\frac{y_i}{\overline{y}}\right)}^{\theta }-1}\right] $$

With representing the numbers of health workforces for *i*th of the population in a sample of size *n*, and $\overline{y}=\frac{1}{n}{\displaystyle \sum_{i=1}^n{y}_i.}$ The parameter *θ* represents the allocation of weight which is given to different population groups, and the most common value of *θ* approaches 0 or 1. When *θ* approaches 0, relatively large shares are distributed to the population groups which have the small number of health workforces, and generalized entropy index is also called the Mean Logarithmic Deviation (MLD). While *θ* approaches 1, the same share is assigned to different groups, and now the generalized entropy index is called Theil index. In present study, we calculated Theil index.

## Result

### Time trend in the number of health workforces in PHC facilities in Jiangsu

**Figure 1:**
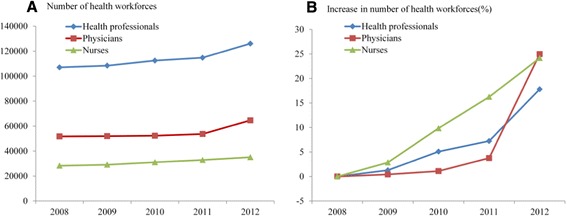
**Time trends in numbers of health workforces in PHC facilities in Jiangsu.** Numbers of health workforces in PHC facilities from 2008 to 2012 are shown **(A)**. Increment ratios in numbers of health workforces compared with those in 2008 are also shown **(B)**.

Even though the base figure of provincial population had been growing since 2008, both the total number of health workforces and the average ratio of health professionals to population had been increasing every year (Figure 1, Table 1). The number of health professionals had been increasing by about 4,800 (4.5%) ever year, and the number of health professionals per 10,000 population has increased from 13.94 to 15.92 in the past five years. From 2008 to 2012, the physician-nurse ratio was about 1:0.54 ~ 0.61 in PHC facilities. That exhibited a decreasing trend before 2011, while it rose obviously in 2012. The physician-health professional ratio was about 1:1.95 ~ 2.15, which showed a similar trend with the ratio of the physician to nurse.

**Table 1:** **Health workforces in PHC facilities in Jiangsu from 2008 to 2012**

**Year**	Population (*10000)	**Health professionals**		**Physicians**		**Nurses**		**Physician/nurse**	**Physician/health professional**
		**No.**	**No./10000**	**No.**	**No./10000**	**No.**	**No./10000**		
2008	7676.50	107026	13.94	51715	6.74	28266	3.68	1:0.55	1:2.07
2009	7724.50	108390	14.03	51934	6.72	29076	3.76	1:0.56	1:2.09
2010	7865.99	112460	14.30	52280	6.65	31045	3.95	1:0.59	1:2.15
2011	7898.80	114769	14.53	53662	6.79	32854	4.16	1:0.61	1:2.14
2012	7919.98	126081	15.92	64629	8.16	35094	4.43	1:0.54	1:1.95

*indicates multiplication.

### Time trend in measures of inequality of health workforces in PHC facilities

**Figure 2:**
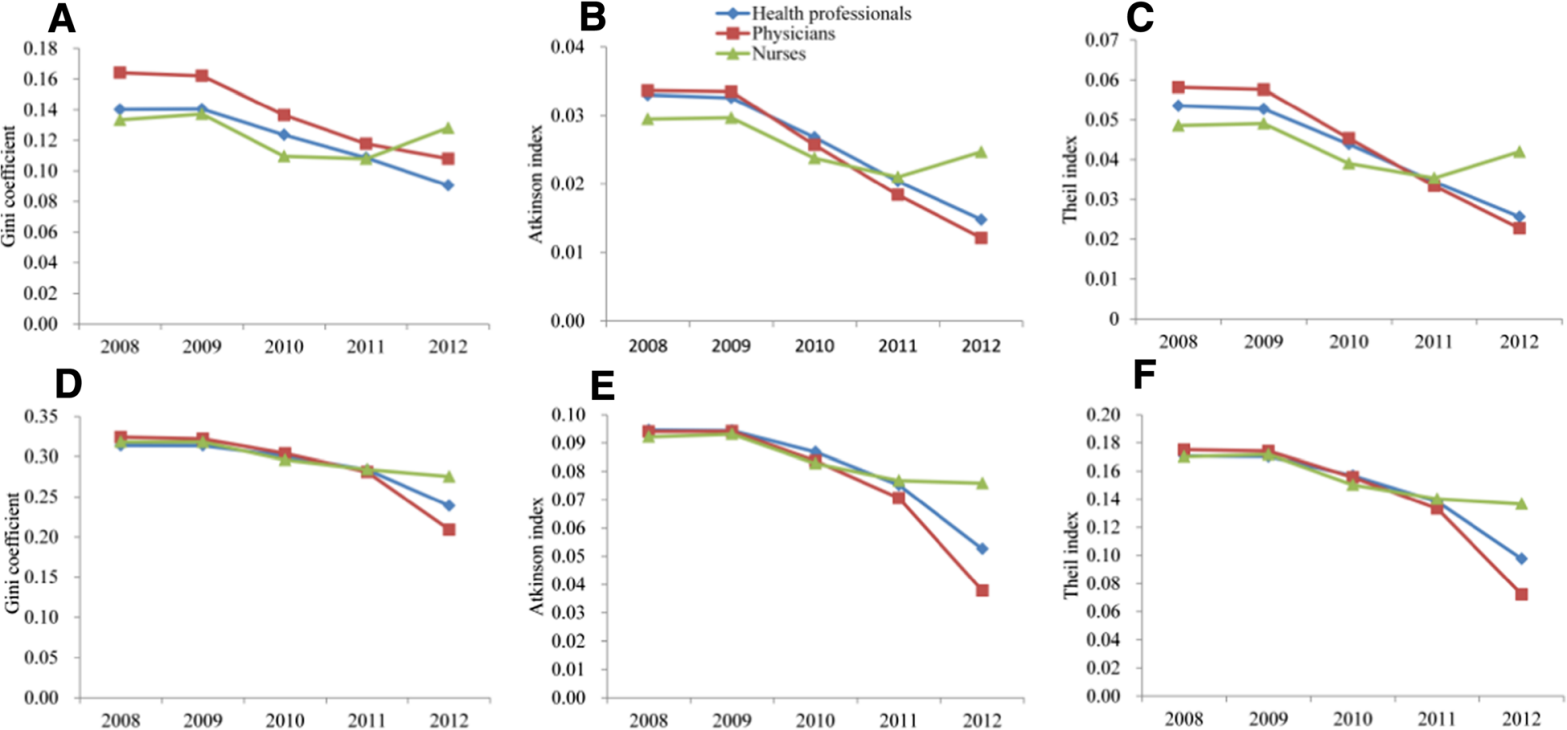
**Trends in Gini coefficient, Atkinson index and Theil index for the distribution of health workforces between 2008 and 2012.** Measures of inequality for health workforces based on population **(A, B and C)** and area **(D, E and F)** are shown.

Three measures of inequality showed similar trends from 2008 to 2012 (Figure 2). From 2008 to 2009, the measures of inequality in the distribution of health workforces improved slightly, while from 2009 to 2012 the measures declined rapidly, especially for the distribution of health professionals and physicians in PHC facilities. For nurses, the three measures of inequality across population declined until 2011, and worsened slightly in 2012.

### Distribution of health workforces in PHC facilities of all cities in Jiangsu in 2012

We analyzed in detail the distribution of health workforces of all cities in Jiangsu in 2012. In terms of population, the mean number of health professionals per 10,000 population for the whole province was 15.92, but this ranged from a low of 4.84 (in M city) to 20.25 (in H city), a 4.2-fold difference (Table 2). The provincial average of the nurse per 10,000 population was 4.43, with a range from 0.92 in M city to 5.92 in H city and a 6.5-fold difference. Analysis of the health professionals based on regions’ classification showed that the central area had the highest number of health professionals and physicians per 10,000 population, including 16.95 and 9.18 respectively, and the highest number of nurses per 10,000 population was in the south of Jiangsu. The north had the lowest number of health professionals (15.46 per 10,000 population) and nurses (7.17 per 10,000 population) among the three regions. The ownership of physician was relative low in the central.

**Table 2:** **Health workforces per 10,000 population and per square kilometer in PHC facilities in each cities and districts of Jiangsu in 2012**

**Category**	Population (*10000)	**Area (km** ^**2**^ **)**	**Health professionals**		**Physicians**		**Nurses**	
			**Population (No./10000)**	**Area (No./km** ^**2**^ **)**	**Population (No./10000)**	**Area (No./km** ^**2**^ **)**	**Population (No./10000)**	**Area (No./km** ^**2**^ **)**
**City**								
A	816.10	6587.00	15.22	1.89	6.88	0.85	4.67	0.58
B	646.55	4627.00	13.76	1.92	6.56	0.92	4.24	0.59
C	856.41	11259.00	17.59	1.34	7.93	0.60	5.21	0.40
D	468.68	4372.00	17.87	1.92	8.73	0.94	5.26	0.56
E	1054.91	8488.00	16.23	2.02	8.19	1.02	4.55	0.57
F	729.73	8001.00	15.45	1.41	8.49	0.77	3.87	0.35
G	440.69	7615.00	16.39	0.95	7.53	0.44	5.44	0.32
H	480.30	10072.00	20.25	0.97	10.41	0.50	5.92	0.28
I	721.63	16972.00	16.23	0.69	10.17	0.43	3.14	0.13
J	446.72	6591.00	17.45	1.18	9.07	0.61	5.11	0.35
K	315.48	3847.00	17.20	1.41	8.93	0.73	5.26	0.43
L	462.98	5787.00	18.84	1.51	10.38	0.83	4.51	0.36
M	479.80	8555.00	4.84	0.27	3.58	0.20	0.92	0.05
**Region**								
Southern	3301.72	27921.00	15.82	1.87	7.70	0.91	4.69	0.55
Central	1639.43	20379.00	16.95	1.36	9.18	0.74	4.39	0.35
Northern	2978.83	54473.00	15.46	0.85	8.11	0.44	4.17	0.23
**Total/average**	7919.98	102773.00	15.92	1.23	8.16	0.63	4.43	0.34

*indicates multiplication.

With respect to area, there was an average of 1.23 health professionals per square kilometer, ranging from 0.27 (in M city) to 2.02 (in E city), a 7.4-fold difference. Among them the physician and nurse occupied 0.63 and 0.34 people per square kilometer. The number of nurses per square kilometer ranged from 0.05 (in M city) to 0.59 (in B city), with an 11.5-fold difference. The number of health professionals per square kilometer (1.87 persons per square kilometer) in the south was more than double the number (0.85 persons per square kilometer) in the north. The south had 0.91 physicians and 0.55 nurses per square kilometer, while the north only had 0.44 physicians and 0.23 nurses per square kilometer.

### Lorenz curves of the distribution of health workforces based on population and area

**Figure 3:**
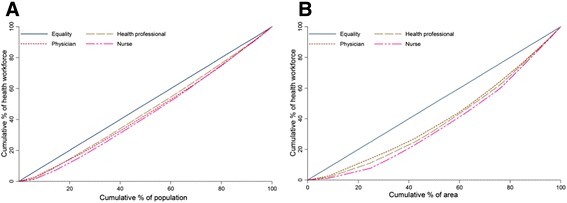
**Lorenz curves of the distribution of health workforce based on population (A) and area (B) in PHC facilities in Jiangsu for 2012.**

Lorenz curves both based on population and area are represented in Figure 3. In 2012, the curves representing the health workforce based on population were closer to the diagonal of equality than the curves based on area. The curve corresponding to the health professional based on population was closer to the diagonal, compared to the curves representing the physician and nurse. On the contrary, the curve representing the nurse based on population or area was the furthest from the diagonal of equality.

### Inequality in the distribution of health workforces of Jiangsu in PHC facilities in 2012

The levels of the Gini coefficient, Atkinson index and Theil index were not comparable since they were constructed in different ways, but the inequality indicators were relative low values of these measures (Table 3). The values of Gini coefficient, Atkinson indices, Theil indices across area were larger than those across population.

**Table 3:** **Measures of inequality of health workforces based on population and area in PHC facilities of Jiangsu in 2012**

**Health workforces**	**Gini coefficient**	**Atkinson index**	**Theil index**
**Based on population**			
Health professional	0.0906	0.0148	0.0256
Physician	0.1081	0.0121	0.0227
Nurse	0.1282	0.0247	0.0420
**Based on area**			
Health professional	0.2389	0.0526	0.0975
Physician	0.2090	0.0378	0.0722
Nurse	0.2753	0.0758	0.1367

## Discussion

The current study was the first provincial study that assessed the trends in the distribution of health workforces in PHC facilities since the latest health reform in China. The results showed that the number of health workforces presented a slow growth from 2008 to 2009, while after 2009 the number of health workforces grew rapidly. Similar trends were found in the measures of inequality for the distribution of health workforces, especially for the distribution of health professionals and physicians between 2008 and 2012. The most potential explanation for these trends in the number and the distribution of health workforces was the health reform in 2009. In 2009, a new wave of reforms was set in motion in China’s health care, in which the proposal to rebuild a good PHC system occupied a central role [24]. If the goal was achieved, primary health care system would play a gate-keeping role in reducing the medical cost burden arising from uncontrolled and irrational use of expensive health services by providing medical care, disease prevention, health promotion and education, rehabilitation and birth control. To achieve these objectives, China’s government had taken several measures in this reform. First of all, there would be 127 billion dollars funded specially to develop infrastructure and human resources in PHC facilities. Secondly, mechanisms for bidirectional referral between PHC facilities and upper-level hospitals would be established to separate their different roles in health care [25]. Finally, PHC system would change the state of dependent on sales of drugs, and governmental subsidies and service charges would become their major income [26]. These policies offered health workforces material guarantee, clarified their job responsibilities and effectively promoted the development of PHC facilities. However, although the number of nurses was rising over the study period, the measures of inequality of nurses worsen in 2012. Similar results have also been concluded in other studies. Isabel and Paula [27], analyzing the equality in geographic distribution of physicians and its evolution in Portugal, concluded that the impact of the growing number of physicians on geographic distribution during the period studies was small. In a study from Japan, Toyabe [28] found that the total number of physicians increased every year in the period from 1996 to 2006, but all three measures of mal-distribution of physicians worsened after 2004. These results implied that a significant increase in the supply of health workforces does not necessarily lead to improvement of the inequality in the distribution of health workforces. On the contrary, the distribution of health workforces may be worsen, because new health workforces may prefer large health facilities rather than small and remote facilities. Further research is needed to determine true reasons for these results.

Although the physician-nurse ratio generally showed a rising trend between 2008 and 2012, the ratio was about 1:0.54 ~ 0.61, which was far below the international standard (1:2 ~ 4). Compared with the international standard, the disproportionality between physicians and nurses in PHC facilities in the province was serious. Many studies reported similar results in China. Zhang et al. [29] investigating the 80 organization and 1494 doctors and nurses in community health service in five city of Jiangsu province, found that the ratio between doctors and nurses was 1:0.76. To evaluate the equity of human resource allocation of community health services, Yao et al. [30] reported that the physician-nurse ratio was 1:0.625 and the proportion of nurses was low. In a comparative study on the number of health workforces in PHC facilities between Anhui and the nation from 2004 to 2010, Luo [12] found that the physician-nurse ratio was about 1:0.4 ~ 0.8 in Anhui, and in nation the ratio was about 1:0.4 ~ 0.6. These results showed that there was a shortage in absolute number of nurses in PHC facilities. Unclear duties of health professionals in PHC facilities may play an important role in this shortage and disproportionality. Unlike many western countries, in China many jobs which should be taken by nurses were done by physicians, especially in PHC facilities. Gong et al. [31] suggested that the international standard that the physician to nurse is 1:2 ~ 4 should be applied in China cautiously. So suitable criteria for China should be established and could not be completely in accordance with the international standard.

Further analysis of the distribution of health workforces in different cities in 2012 revealed that there were 6.5-fold variation in the number of nurses per 10,000 population and 11.5-fold variation in the number of nurses per square kilometer. These may be why the curves representing nurses were the furthest from the diagonal of equality. In addition, the average number of health workforces per population and square kilometer in the north was lower than other two regions. This might be related to regional social and economic development. Assessing the pure and social disparities in the distribution of dentists across the provinces in Iran, Kiadaliri et al. [32] concluded that there were strong positive correlations between density of dentists and better social rank. While the economic development of the south and the central area were higher than the north and the local governments might invest more to develop the PHC service. This suggested that the provincial government policy should be relatively inclined to the northern to keep equality in PHC service.

Health workforces from PHC facilities were equitably distributed based on population in the province between 2008 and 2012, while based on area health workforces distributed relatively equitably. When all the measures of inequality indices were examined, these became apparent. Similar results have been concluded in the past in other districts in China, such as the Wuhan, [33] Xizang, [34] and Hubei [30]. Yao et al. [30] thought that the reason of these differences was that when the governments formulated health policies and strategies, they always referred per capita possession as standard of planning and construction of PHC facilities. If the allocation of health resources across area was neglected, it would be not convenient for people to get equal access to primary health care services and increase the workload of health workforces [33]. For the general layout planning of PHC facilities, the government needs to give consideration to local geographical factors to promote service efficiency of PHC facilities.

One limitation of this study was that the measures of equality of health workforces were not adjusted by health status, health service needs and health service utilization. Inequality indicators may be affected by these factors. In addition, due to acceleration in the intergration of the rural and urban areas, the distribution of health workforces was analyzed throughout the province not in urban and rural regions separately. We could not decompose inequality indices into the contribution of its determinants to further evaluate the distribution of health workforces. Therefore, the inequality in the distribution of health workforces derived from the intra- and inter-cities needs to be further elucidated in future studies.

## Conclusion

The number of health workforces in PHC facilities in Jiangsu increased every year from 2008 to 2012, and the inequality in the distribution of health workforces showed a decline trend during that period. The health reform in 2009 might play an important role in these trends. However, there was disproportionality between physicians and nurses in PHC facilities in the province, which was mainly due to a shortage in absolute number of nurses. Many initiatives for primary health nurses should be implemented, including generous admission policies, recruitment of trained nurses, and more learning opportunities. The distribution of health workforce based on population was more equal than based on area. Health statistical data provide an economic and useful way to evaluate the impact of health reform on the primary health care. If China solved the problems of ‘difficult access to medical services and expensive medical cost’ by the primary health care system, she would once again become a good example for other countries.

## Abbreviations

PHC: Primary health care
CHOs: Community health service organizations
THCs: Township health centers
NFHPC: National Health and Family Planning Commission
DASP: Distribution analysis stata package
